# Recent advances in idiopathic bilateral vestibulopathy: a literature review

**DOI:** 10.1186/s13023-019-1180-8

**Published:** 2019-08-19

**Authors:** Chisato Fujimoto, Masato Yagi, Toshihisa Murofushi

**Affiliations:** 1 Department of Otolaryngology, Tokyo Teishin Hospital, 2-14-23, Fujimi, Chiyoda-ku, Tokyo, 102-8798 Japan; 2 Department of Otolaryngology and Head and Neck Surgery, Graduate School of Medicine, The University of Tokyo 7-3-1, Hongo, Bunkyo-ku, Tokyo, 113-8655 Japan; 3 Department of Otolaryngology, Teikyo University School of Medicine Mizonokuchi Hospital 5-1-1, Futako, Takatsu-ku, Kawasaki, Kanagawa 213-8507 Japan

**Keywords:** Bilateral vestibulopathy, Posture, Vertigo, Vestibule, Vestibular function tests

## Abstract

**Background:**

Idiopathic bilateral vestibulopathy (IBV) is an acquired bilateral peripheral vestibular dysfunction of unknown etiology, with persistent unsteadiness but without sensorineural hearing loss (SNHL) other than age-related hearing loss (ARHL).

**Main text:**

The prevalence of IBV is unknown. The most common symptom is persistent unsteadiness, particularly in darkness and/or on uneven ground. The other main symptom is oscillopsia during head and body movements. IBV is neither associated with SNHL, except for ARHL, nor any other neurological dysfunction that causes balance disorders. The clinical time course of IBV can generally be divided into two main types: progressive type and sequential type. The progressive type involves gradually progressive persistent unsteadiness without episodes of vertigo. The sequential type involves recurrent vertigo attacks accompanied by persistent unsteadiness. Originally, IBV was found to exhibit bilateral dysfunction in the lateral semicircular canals (LSCCs) and the superior vestibular nerve (SVN) system. However, recently, with the development of more sophisticated vestibular function tests of the otolith organs and vertical semicircular canals, it has been revealed that IBV involves peripheral vestibular lesions other than those already identified in the LSCC and the SVN system. Furthermore, novel subtypes of IBV that do not involve bilateral dysfunction of the LSCC and/or the SVN system have been proposed. Therapeutically, exercise-based vestibular rehabilitation in adult bilateral vestibulopathy (BVP) patients has resulted in improved gaze and postural stability moderately. There are several technical approaches for the treatment of BVP such as vestibular implants, sensory substitution devices and noisy galvanic vestibular stimulation.

**Conclusions:**

Combined use of various vestibular function tests, including recently developed tests, revealed the diversity of lesion sites in IBV. Further studies are required to determine the therapeutic effects of the technical approaches on IBV.

## Background

Idiopathic bilateral vestibulopathy [(IBV), ORPHA 171684, ICD-10 H81.8] [1] is an acquired bilateral peripheral vestibular hypofunction of unknown etiology, which was first proposed by Baloh et al. in 1989 [1]. Synonyms of IBV include bilateral idiopathic loss of vestibular function (BILVF) [2], idiopathic bilateral vestibular loss [3], idiopathic bilateral vestibular hypofunction [4] and idiopathic bilateral loss of vestibular function [5]. The most common symptom of IBV is persistent unsteadiness, particularly in darkness and/or on uneven ground [1]. The other main symptom is oscillopsia during head and body movements [1]. IBV is neither associated with sensorineural hearing loss (SNHL), except for age-related hearing loss (ARHL), nor any other neurological dysfunction that causes balance disorders [1]. Originally, IBV was found to exhibit bilateral dysfunction in the lateral semicircular canals (LSCCs) and the superior vestibular nerve (SVN) system, as shown by caloric and rotation tests [1]. Later, the development of vestibular function tests such as the vestibular evoked myogenic potential (VEMP) test [6–9] and the video head impulse test (vHIT) [10] enabled more detailed assessments of the function of the otolith organs and vertical SCCs (VSCCs). Consequently, it was found that IBV could involve peripheral vestibular lesions other than those of the LSCC and the SVN system [5, 11–17]. Furthermore, novel subtypes of IBV that do not indicate bilateral dysfunction of the LSCC and/or the SVN system have also been proposed [11, 13–16].

## Main text

### Epidemiology

The true prevalence of IBV is not known yet due to insufficient data. A previous study based on the 2008 United States National Health Interview Survey Balance and Dizziness Supplement reported that the prevalence of bilateral vestibulopathy (BVP) was 28/100,000 adults [18]. However, the diagnosis of BVP in this study was survey-based and was not made by vestibular function tests. It has been said that the cause of 20–50% of cases of BVP remains unknown [19–23], but this percentage includes patients with SNHL, so the percentage of IBV cases would be much smaller. On the other hand, novel subtypes of IBV, which do not show bilateral dysfunction in the LSCC and the SVN system, have been recently reported [11, 13–16]. If these subtypes are included, the prevalence could be higher.

### Etiology and pathogenesis

IBV by definition does not have a clear etiology or pathogenesis. In a previous immunological study, sera from IBV patients for antibodies against the inner ear was screened [24]. IgG antibodies against the SCCs, saccule and utricle were detected in 66% of the IBV patients by immunostaining on rat inner ear tissue sections, as would be expected for human tissues. However, autoantibodies against the cochlea were detected in 25% of the IBV patients without hearing loss, and the anti-vestibular autoantibody titer varied substantially in the IBV patients in spite of their severe vestibular dysfunction. Therefore, autoantibodies against the vestibular end organs may not be pathogenic, but an epiphenomenon in IBV. Mitochondrial 12S rRNA susceptibility mutations have been shown in some IBV patients, although their pathogenic role in vestibular dysfunction remains unclear [25]. Another study reported endothelial dysfunction in a case of bilateral vestibular hypofunction with unknown cause, which was evaluated by functional assessment of endothelium-dependent vasodilation using high-resolution ultrasonography and the blood level analysis of soluble intercellular adhesion molecule-1 [26]. However, BVP with unknown cause in this study included cases with hearing loss, and the characteristics of the idiopathic cases in this study may be different from those of IBV that do not have SNHL other than ARHL. Another recent study reported that the percentage of migraine among BVP patients with unknown cause was significantly higher than that among BVP patients with a definite or probable etiology, suggesting the association between migraine and BVP with unknown cause [20]. However, even in this study, BVP with unknown cause included the cases with hearing loss and, again, the characteristics of the idiopathic cases in this study may be different from that of IBV.

The involvement of multiple factors in IBV is also indicated by the fact that some cases involve vertigo attacks, while others do not [1]. Vestibular neuritis is clinically characterized by acute unilateral vestibular dysfunction. Infection [27, 28], ischemia [29], and autoimmunity [30] have all been hypothesized for the etiology of vestibular neuritis, but its etiology remains controversial [31]. If vestibular neuritis is considered to be an acute peripheral vestibular dysfunction of unknown cause, bilateral sequential vestibular neuritis can be regarded as a kind of IBV [3, 32, 33]. Recently, novel possible etiologies of BVP such as amiodarone medication [34–36] and exposure to military jet fuel [37] have been reported. Therefore, the ratio of IBV in BVP might decrease in the future.

### Clinical features

#### Symptoms

The most common symptom of IBV is persistent unsteadiness [1]. In darkness and/or on uneven ground, IBV patients become more unstable because of their increased dependence on vestibular input to maintain their balance in such situations. The other main symptom is oscillopsia during head and body movements [1]. These symptoms are generally observed in BVP patients, but there are some BVP patients who do not have oscillopsia [1, 3]. A rare IBV case described two types of oscillopsia, one induced by head movements, the other induced by changing pressure in the external auditory canal [38]. Another visual symptom of BVP is decreased visual acuity in dynamic conditions. Although some BVP patients with unknown cause complain of hearing loss, IBV is generally limited to cases that do not have SNHL other than ARHL [1]. It has been reported that 20–60% of IBV patients have episodes of vertigo [1, 3, 16]. IBV is not associated with any other obvious neurological dysfunction that could cause a balance disorder.

#### Clinical time course

The clinical time course of IBV is generally divided into two main types: progressive type and sequential type [1]. The progressive type involves gradually progressive persistent unsteadiness without episodes of vertigo. The sequential type involves recurrent vertigo attacks accompanied by persistent unsteadiness. The duration of vertigo attacks in the sequential type varies from patient to patient. Some patients have a history of recurrent vertigo lasting more than 1 day, as is typical in vestibular neuritis, while others suffer from repetitive vertigo attacks lasting for 30 min up to several hours [16]. Recently, it was reported that a small proportion of IBV patients had only a single episode of a vertigo attack [16, 39]. Patients with the single-attack type show persistent unsteadiness following the vertigo attack.

### Diagnosis

#### Diagnostic criteria of BVP

With regard to BVP, the diagnostic criteria were recently released by the Classification Committee of the Barany Society (see Appendix [40]). These criteria include only BVP cases with a very severe LSCC dysfunction. On the other hand, the cases with dysfunction of the VSCC or otolith organs are not included. Thus, cases meeting these criteria may reflect an aspect of BVP but may not be appropriate for obtaining a complete view of BVP. Therefore, in this paper, in order to put IBV in a broad perspective, IBV is characterized as acquired bilateral peripheral vestibular dysfunction of unknown cause, with persistent unsteadiness, without SNHL other than ARHL.

#### Methods of diagnosis of IBV

As a baseline for diagnosis, IBV is characterized by acquired bilateral peripheral vestibular dysfunction with unknown cause, with persistent unsteadiness, without SNHL other than ARHL [1]. Oscillopsia during head and body movement is also a main symptom of IBV, but the presence of oscillopsia is not required for the diagnosis of IBV. IBV is not associated with any other neurological dysfunction that causes balance disorders [1].

Revealing bilateral dysfunction of the LSCC and/or the SVN system using the caloric test and the rotation test, both of which assess the vestibulo-ocular reflex (VOR) at LSCC plane, has historically been the most common method of diagnosing IBV [1–5, 24, 38, 41]. Some studies regarded abnormalities in both the caloric test and rotation test as indicating bilateral vestibular dysfunction [1, 2, 4, 5, 41], while other studies regarded abnormalities in either the caloric test or the rotation test as bilateral vestibular dysfunction [3, 24, 38]. There have also been studies which only performed caloric testing, and regarded caloric abnormalities as bilateral vestibular dysfunction in the absence of rotation testing [12, 14, 17, 42]. Behind this background, evaluation of vestibular dysfunction has been historically performed by the caloric test or rotation test, both of which assess the vestibulo-ocular reflex (VOR) at LSCC plane.

The development of VEMP testing made it possible to measure function in the otolith organs [6–9]. The cervical VEMP (cVEMP) test measures the function of the saccule and inferior vestibular nerve (IVN) system [6, 8, 9], while the ocular VEMP (oVEMP) test measures the function of the utricle and SVN system [7, 8]. Investigation of vestibular function using VEMP testing in IBV patients revealed that some IBV patients showed abnormal VEMPs [5, 12, 14–17, 41]. In addition, novel subtypes of IBV, which do not show bilateral dysfunction in the LSCC and/or the SVN system, were reported [11, 13–16]. First, an IBV subtype with bilateral absence of cVEMPs in the presence of normal caloric responses was reported [15]. Then, it was reported that some BVP patients showing abnormal caloric responses on one side and abnormal cVEMPs on the contralateral side could be categorized into a novel IBV subtype [13]. These studies suggest that the lesions involved in IBV occur not only in the SVN system but also in the IVN system.

Since a decrease in VOR gain demonstrated by vHIT or scleral-coil HIT at the LSCC plane is included in the diagnostic criteria for BVP [40], some of the diagnoses of BVP due to an abnormality in vHIT are considered to be IBV. If limited to reports of IBV alone, there are some previous reports that conducted HIT for IBV patients [3, 5, 25], and one report confirmed that IBV patients showed both bilaterally-reduced caloric responses and bilaterally-reduced VOR gain in HIT [25]. Recently, vHIT has become more widely available, allowing easy measurement of the VOR not only at the LSCC plane but also the VSCC plane [10]. It has been reported that anterior SCC function is less deteriorated than the other SCC functions in BVP cases with unknown cause [11, 22]. In the BVP cases with unknown cause in these reports, cases with bilateral SNHL were excluded, but it was not described if unilateral SNHL was present. The results derived from the idiopathic cases in these reports may be different from those in IBV. The diversity of lesion sites in BVP, including IBV, becomes clear through combined use of caloric testing and/or vHIT, cVEMPs and oVEMPs [11].

The main problem for the establishment of an understanding of otolithic involvement in BVP is the accuracy of assessment of bilateral losses of otolith function. While assessment of unilateral dysfunction of the otolith organs is quite easy, judgement of bilateral dysfunction of the otolith organs is not easy. In previous reports of IBV, bilaterally absent cVEMP responses was regarded as bilaterally abnormal cVEMPs [14–16]. The criteria for defining bilateral otolith dysfunction should be clearly established.

### Examination findings other than peripheral vestibular function tests

#### Postural control

A previous study assessed postural stability using foam posturography in IBV patients according to whether the SVN systems, the IVN systems or both of the vestibular nerve systems were affected [14]. While function of the SVN systems was examined by caloric testing, function of the IVN system was determined by cVEMP testing. IBV significantly affects static postural stability and the instability was more severe in patients with damage to both of the vestibular nerve systems in comparison with isolated damage to either the SVN systems or the IVN systems. Residual function in the spared vestibular nerve system might play an important role in the postural stability of IBV patients.

#### Vergence eye movements

A previous study investigated vergence eye movements in IBV patients, by using a light emitting diode display with targets along the median plane [5]. Convergence eye movements in IBV patients showed a significantly disturbed trajectory, lower mean velocity, and higher amplitude saccadic intrusions than in control subjects.

#### Motion perception

A previous study reported the abnormality of vestibular perceptual thresholds in IBV patients [4]. Subjects were seated on a motion platform, and the perceptual thresholds were measured for 4 motion paradigms: yaw rotation (testing the LSCCs), interaural translation (testing the utricles), superior-inferior translation (testing the saccules), and roll tilt (testing the VSCCs and the otolith organs). Perceptual thresholds were abnormally elevated in IBV patients for yaw rotation at all frequencies and for interaural translation at the lower frequencies.

#### Dynamic visual acuity tests

In BVP patients, gaze stabilization fails and can lead to decrease in visual acuity during head movements. There are various testing paradigms to evaluate dynamic visual acuity in BVP patients, such as reading an optotype chart, during fast head movements [43], passive head shaking [3, 19], or walking on a treadmill [44]. These tests are helpful to demonstrate decreased dynamic visual acuity in BVP patients.

#### Others

It was reported that BVP led to a significant decrease in the gray matter mid-hippocampal volume and posterior parahippocampal volume [45]. BVP also led to a higher spatial anxiety revealed by self-reported questionnaires and a delayed spatial learning performance revealed by a virtual Morris Water Maze Task [45]. However, in this report, IBV patients were only a small portion of the BVP patients studied, and care should be taken of the interpretation of the results.

Recently, a systematic review about the impact of BVP on spatial and non-spatial cognition was performed [46]. In this review, strong evidence existed that BVP patients suffer from impaired spatial and non-spatial cognition. However, conclusions on the association between cognitive performance and vestibular dysfunction were drawn without considering hearing loss as a possible cause of the cognitive impairment. As IBV patients do not show SNHL other than ARHL, assessing the cognitive function in IBV patients may lead to a more accurate assessment of the influence of the vestibular system on cognitive function.

### Treatment

The treatment of IBV has not been distinguished so far from that of standard BVP. Here, we describe the treatment of BVP.

The effect of vestibular rehabilitation on BVP remains controversial due to conflicting results in previous reports. One systematic review of the effects of vestibular rehabilitation on adult BVP patients revealed moderate evidence for improved gaze and postural stability [International Classification of Functioning, Disability and Health (ICF) - Body Functions] following exercise-based vestibular rehabilitation [47].

There are several technical approaches for the treatment of BVP. Vestibular implants stimulate the peripheral vestibular nerve through electrical pulses, and has been proposed as a candidate for the treatment of BVP [48–50]. Restoration of the VOR has been revealed in preliminary human studies [50]. This treatment requires surgery that has potential risks including hearing loss, hence it must be carefully considered, especially in the treatment of IBV patients who do not have SNHL. Sensory substitution devices have been developed to substitute for the loss of vestibular feedback by providing concurrent tactile or auditory stimulation [51–54]. Previous studies reported the ameliorating effect of electrotactile or auditory vestibular substitution on balance control in BVP patients [51–54]. Noisy galvanic vestibular stimulation (nGVS) is a procedure that applies zero-mean current noise to the vestibular end organs and their afferent nerves through electrodes placed bilaterally over the mastoid process [42, 55–58]. An imperceptible level of nGVS improves postural and gait stability during the stimulus in BVP patients [55, 59, 60]. The suggested mechanism underlying these effects is stochastic resonance, in which the existence of an optimal amount of noise can enhance the detection of subthreshold signals in non-linear systems [61, 62]. Recently, it was reported that nGVS leads to a sustainable improvement in postural stability in BVP patients, an effect that lasted for several hours, even after the cessation of the stimulus [63]. For these technical approaches, further studies are needed to increase the evidence level of their therapeutic effects.

## Conclusions

Combined use of various vestibular function tests, including recently developed tests, revealed the diversity of lesion sites in IBV. Further studies are required to determine the therapeutic effects of the technical approaches such as vestibular implants, sensory substitution devices and nGVS on IBV.

## Data Availability

Not applicable.

## Appendix

Diagnostic criteria were recently released by the Classification Committee of the Barany Society:

A. Chronic vestibular syndrome with the following symptoms:

1. Unsteadiness when walking or standing plus at least one of 2 or 3.
2. Movement-induced blurred vision or oscillopsia during walking or quick head/body movements.
  and/or.
3. Worsening of unsteadiness in darkness and/or on uneven ground.

B. No symptoms while sitting or lying down under static conditions.

C. Bilaterally reduced or absent angular VOR function documented by:

1. Bilaterally pathological horizontal angular VOR gain < 0.6, measured by the vHIT or scleral-coil technique
2. and/or.
3. Reduced caloric response (sum of bithermal maximum peak slow phase velocity on each side < 6° /sec)and/or.
4. Reduced horizontal angular VOR gain < 0.1 upon sinusoidal stimulation on a rotatory chair (0.1 Hz, Vmax = 50° /sec) and a phase lead > 68 degrees (time constant < 5 s).

D. Not better accounted for by another disease.
